# EVALUATION OF CHILDREN WITH RADIAL NECK FRACTURES TREATED WITH FLEXIBLE INTRAMEDULLARY NAIL

**DOI:** 10.1590/1413-785220162402154788

**Published:** 2016

**Authors:** Isabella da Costa Gagliardi, Guilherme Grisi Mouraria, Bruno Funayama, Fernando Kenji Kikuta, Márcio Alves Cruz, Américo Zoppi

**Affiliations:** 1. Universidade Estadual de Campinas (UNICAMP), Faculdade de Ciências Médicas, Department of Orthopedics and Traumatology. Campinas, SP, Brazil

**Keywords:** Fractures, bone, Radius fractures, Child, Fracture fixation, intramedullary

## Abstract

**Objective:**

: To evaluate the results of displaced radial neck fractures in children trated surgically with flexible titanium intramedullary nails.

**Method:**

: This is a retrospective study of five cases of radial neck fractures with displacement greater than 30° fixed with flexible intramedullary nails. Patients were evaluated regarding functional outcome through range of motion and the Mayo Elbow Performance Score (MEPS), as well as radiographic exams.

**Results:**

: Five patients, with a mean age of 8.4 years were assessed, during a mean post-operative follow up of 12.2 months. Open reduction was necessary in three cases with major displacement. At the end of the follow up, 80% of the patients had excellent results, 20% good results, and all fractures healed. As complications we observed: heterotopic ossification, superficial infection and radial head necrosis.

**Conclusions:**

: In spite of the small sample, our results with flexible titanium intramedullary nails were similar to the current literature, with good functional outcomes. Level of Evidence III, Retrospective Study.

## INTRODUCTION

Radial neck fractures in children are rare and account for about 1% of all pediatric fractures and 5-10% of elbow fractures in children.^1^^,^ 2 The age group with the highest prevalence is between 8 and 12 years old. The main injury mechanism is a fall with the elbow in hyperextension, the forearm supinated and valgus force causing radial head compression against the capitulum.^3^^,^ 4

O'Brien classified radial neck fractures in children as types I, II and III. Type I fractures are those with deviation less than 30°, type II with deviation between 30-60° and type III, deviation greater than 60°.^5^

The deviation of the fracture and the patient's age are factors that determine the type of treatment.^5^^-^ 8 Type I fractures are treated conservatively, type III are treated surgically and type II can be treated conservatively or surgically, depending on the age of the patient. However, there is a current trend to perform surgical treatment.^2^^,^ 9

Several techniques for surgical treatment of displaced radial neck fractures are described in the literature, including percutaneous reduction with Kirschner wires described by Métaizeau et al.,^1^ use of flexible intramedullary nails and open reduction with or without internal fixation.^2^^,^ 8 ^,^ 10 ^-^ 13

Fractures treated surgically, but without open reduction, have better functional outcomes than those treated with direct visualization of the fracture site.^2^^,^ 7 ^-^ 9 ^,^ 14 ^,^ 15

We performed a retrospective study evaluating clinical and radiographic results of children with radial neck fractures with deviation greater than 30° treated with titanium elastic intramedullary nail.

## MATERIALS AND METHODS

This is a retrospective study, a survey of a series of five cases of radial neck fractures treated at *Hospital Estadual de Sumaré da Faculdade de Ciências Médicas da Universidade Estadual de Campinas* (*Unicamp*), between 2011-2014.

Fractures were ranked according to O'Brien classification.(Table 1) The deviation is determined by the angle between a line perpendicular to the radial head articular surface and a line in the long diaphyseal axis in the initial radiographs.

**Table 1.** O'Brien classification for radius neck fractures.

**Type**	**Deviation angle**
I	<30º
II	30-60º
III	>60º

Patients with radial neck fracture and open proximal physeal plate of the radius, O'Brien types II and III, which were surgically treated with flexible titanium intramedullary nail were included in the study.

Patients with smaller deviations (O'Brien type I), patients with radial dysplasia, previous fracture sequel, less than 6 months follow-up or those whose data was not properly included in the medical records were excluded from the study.

**Figure 1.**
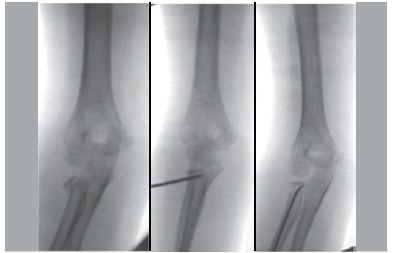
Reduction with the aid of Kirschner wire and osteosynthesis with flexible nail.

The patients were operated in the supine position under general anesthesia. A closed reduction maneuver with varus stress, as described by Patterson, was performed.^16^ Upon failure of closed reduction, a flexible nail was introduced at the distal radius region (without compromising the integrity of the physeal plate) and a reduction of the fracture was attempted, with the aid of the nail as in Métaizeau's technique. In case this procedure was not effective, we used a Kirschner wire as a "joystick", as described by Böhler^12^, and then the rod was introduced as previously described. (Figure 1) Only when the previous maneuvers failed, fracture reduction was performed in open manner. Even in cases of direct reduction, we used the rod as fracture fixation. In all cases, the flexible rod was introduced by the distal radius to exceed the fracture at the proximal radial region without leaving it intraarticular. The flexible rod was removed in all cases after at least three months from surgery.

For functional evaluation of the elbow, the Mayo Elbow Performance Score (MEPS) was used. (Table 2) We also evaluated the arc of the elbow motion. The radiographic alignment and consolidation were assessed by plain radiographs in the incidences in anteroposterior and profile views.

**Table 2.** Mayo elbow performance score (MEPS).L

**Score**	**Result**
90-100	Excellent
75-89	Good
60-74	Regular
<60	Poor

The study was approved by the local Ethics Committee under protocol number 1.245.031.

## RESULTS

Five children were evaluated with a mean age of 8.4 years old (mean 8.4 ± 1.3 years old). Three (60%) were female and two (40%) were male. The left elbow was injured in three cases (60%) and the right in two (40%).

The initial average deviation of the fracture was 60 ± 16°. Open reduction was necessary in three cases, because non-reduction and closed reduction according to Böhler and Métaizeau was not feasible.^1^^,^ 11 ^,^ 12

The mean follow-up was 12.1±7.0 months. All patients evolved with fracture consolidation and underwent removal of the rod (with at least 3 months postoperatively). No cases evolved with deep infection or postoperative neurological deficit.

The average arc movement after the withdrawal of the rod was 138.4 ± 8.3° flexion, 4.0 ± 2.0° extension, 55.4 ± 34.8° pronation and 72.0 ± 33.5° supination. (Table 3) Loss of range of motion occurred mainly in pronation.

**Table 3.** Demographic data and functional assessment of patients after removal of the nail.

	**Age**	**Reduction**	**Gender**	**Flexion**	**Extension**	**Supination**	**Pronation**	**MEPS**
Patient 1	9	Open	Fem	125	-5	85	22	100
Patient 2	7	Closed	Fem	145	-5	90	80	85
Patient 3	9	Open	Masc	132	0	5	5	100
Patient 4	7	Closed	Masc	145	-5	90	80	100
Patient 5	10	Open	Fem	145	-5	90	90	100
Mean ± St. Dev	8.4 ± 1.3			138 ± 8.3	-4 ± 2	72 ± 33.5	55.4 ± 34.8	97 ± 3

The functional evaluation of the elbow was taken by score of MEPS, values ​were between 85 and 100, with an average value of 97 ± 6 points. (Table 3) Postoperative loading angle was 11.5 ± 2.1°.

**Figure 2.**
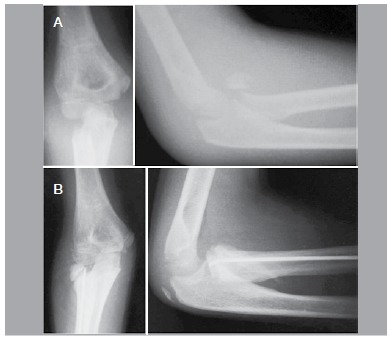
(A) Preoperative x-ray; (B) Postoperative x-ray with signs of osteonecrosis of O'Brien type III fracture.

Regarding complications, there was one case of heterotopic ossification without clinical repercussions, such as decreased range of motion, one case of superficial infection, treated with oral antibiotics and one case of osteonecrosis, which evolved with decreased elbow range of motion. (Figure 2) 

## DISCUSSION

Five children with radial neck fractures were evaluated. There was a higher prevalence of females and the average age was 8.4 years old, which is in accordance with data from literature.^7^^-^ 9 ^,^ 17 ^,^ 18 There was a higher prevalence of fractures in women, but there is no agreement on the literature regarding the most affected gender.^2^^,^ 3 ^,^ 9

There is no consensus regarding surgical treatment in O'Brien type II fractures, however there is a current trend in the literature to perform operative treatment of these injuries. In O'Brien fractures type III, surgery is the treatment choice. The average deviation of the study was 60°, with a minimum angle of 41°.

Numerous surgical techniques have been proposed for the treatment of these fractures, such as closed reduction and fixation with wires or intramedullary nail, percutaneous "joystick" for reduction (Böhler's technique), indirect reduction by intramedullary manipulation of the radial head and direct reduction with arthrotomy.^1^^,^ 2 ^,^ 7 ^-^ 15 Percutaneous reduction methods show better results when compared to open reduction.^2^^,^ 7 ^-^ 9 ^,^ 15 Among them there is the intramedullary fixation technique described by Métaizeau et al.^1^^,^ 11 It preserves the lateral periosteal and, thus, the vascular supply of the radial proximal epiphysis. It is a minimally invasive technique, with good results reported in the literature, by combining advantages of a closed reduction of the fracture with good stability throughout the consolidation. The technique employed in this study follows the same principle, but uses titanium rod for fixation. The use of the rod to aid fracture reduction and fixation is described in the literature and showed good result funcional.^7^^-^ 10 ^,^ 14 Open reduction, widely used in the past, is currently restricted to cases of comminuted fracture or failure of closed reduction or percutaneous reduction. The main causes of this form of intervention are the interposition of annular ligament between the neck and head of the radius and fractures with deviation greater than 60°. In our series, we obtained closed reduction in both cases, as most patients had large deviations, which prevented the percutaneous technique. The average fracture deviation in cases of open reduction was 70±17.5°, showing a large initial deviation. The difficulty to perform closed reduction in cases of deviation greater than 60° is in accordance with the literature.^1^^,^ 2 ^,^ 8 ^,^ 9 ^,^ 14 ^,^ 15

The postoperative functional outcome of patients undergoing osteosynthesis with rod was evaluated by the MEPS score. All patients had excellent or good results, which is in line with the literature.^1^^,^ 7 ^,^ 9 Only one case progressed with significant limitation of range of motion, despite having good MEPS score. This patient had initial deviation of 90° (Figure 2) and developed radial head osteonecrosis. Osteonecrosis is closely associated with arthrotomy, since all epiphyseal region of the radial head is surrounded by articular cartilage. Thus, vascularization is ensured only by the periosteum. Moreover, fractures with large deviations associated to arthrotomy can damage circulation.^2^^,^ 6 ^,^ 9 ^,^ 19 Osteonecrosis is closely related to a decreased range of motion of the elbow.^8^

There was one case of heterotopic ossification without clinical significance, because the patient evolved with a good MEPS score and functional range of motion. The heterotopic ossification secondary to proximal radial fractures usually do not evolve with decreased range of motion of the elbow.^19^

The postoperative complications seen in our case series, such as avascular necrosis of the radial head and heterotopic ossification, are in accordance with the literature, especially in fractures with large deviations (O'Brien type III) and approaches with direct visualization of the fracture focus.^1^^,^ 2 ^,^ 7 ^-^ 9 ^,^ 14 ^,^ 15

The small number of patients was the main limitation of this study, which can be justified by the low prevalence of cases. Nevertheless, the work results agreed with the literature and may contribute to the demonstration of the technique, its difficulties and potential complications.

## CONCLUSION

Surgical treatment of radial head fractures in children with flexible intramedullary nail provided good functional outcome, being a good option for treating this injury.
